# The Role of Allergen-Specific Immunotherapy in ENT Diseases: A Systematic Review

**DOI:** 10.3390/jpm12060946

**Published:** 2022-06-09

**Authors:** Elena Cantone, Stefania Gallo, Sara Torretta, Aikaterini Detoraki, Carlo Cavaliere, Claudio Di Nola, Luca Spirito, Tiziana Di Cesare, Stefano Settimi, Daniela Furno, Lorenzo Pignataro, Eugenio De Corso

**Affiliations:** 1 Department of Neurosciences, Reproductive and Odontostomatologic Sciences, Unit of Ear, Nose and Throat, Federico II University, 80131 Naples, Italy; elena.cantone@unina.it (E.C.); claudio.dinola@unina.it (C.D.N.); 2 Head and Neck Department, ENT Section, AOU Federico II University, 80131 Naples, Italy; 3 ENT Clinic, Ospedale di Circolo e Fondazione Macchi, ASST Sette Laghi, 21100 Varese, Italy; stefania.gallo@me.com (S.G.); lu.spiri@gmail.com (L.S.); 4 UPLOAD (Upper and Lower Airways Inflammatory Diseases) Research Center—University of Insubria, 21100 Varese, Italy; 5 Fondazione IRCCS Ca’ Granda Ospedale Maggiore Policlinico, 20122 Milan, Italy; sara.torretta@gmail.com (S.T.); lorenzo.pignataro@policlinico.mi.it (L.P.); 6 Department of Clinical Sciences and Community Health, University of Milan, 20122 Milan, Italy; 7 Department of Internal Medicine, Clinical Immunology, Clinical Pathology and Infectious Diseases, Division of Internal Medicine and Clinical Immunology, AOU Federico II University, 80131 Naples, Italy; caterina.detoraki@gmail.com; 8 Department of Sensory Organs, Sapienza University of Rome, 00185 Rome, Italy; carlocavaliere1985@gmail.com; 9 Department of Head Neck and Sensory Organs, Catholic University of the Sacred Heart, 00168 Rome, Italy; tizianadicesare90@gmail.com (T.D.C.); daniela.furno1@gmail.com (D.F.); 10 Unit of Otorhinolaryngology Head and Neck Surgery, Fondazione Policlinico Universitario A. Gemelli IRCCS, 00168 Rome, Italy; eugenio.decorso@gmail.com

**Keywords:** AIT, SCIT, SLIT, immunotherapy, LAR, chronic rhinosinusitis, otitis, adenotonsillar disease, personalized medicine, precision medicine

## Abstract

Previous studies have demonstrated that both subcutaneous (SCIT) and sublingual specific immunotherapy (SLIT) are effective in treating allergic rhinitis (AR). Further studies have evaluated the efficacy of allergen-specific immunotherapy (AIT) on different ear, nose, and throat (ENT) manifestations, in which allergy might have an etiopathogenetic role, such as local allergic rhinitis (LAR), rhinosinusitis (RS), otitis media (OM), and adenotonsillar (AT) disease. Nevertheless, the management of allergy in ENT diseases is still debated. To the best of our knowledge, this is the first systematic review assessing the efficacy of AIT in ENT diseases aside from AR. Literature data confirmed that AIT might be an effective therapeutic option in LAR, although its effect is restricted to studies with short-term follow-up. Furthermore, previous research demonstrated that AIT may improve symptoms and surgical outcomes of chronic rhinosinusitis when used as an adjunctive treatment. Few studies supported the hypothesis that AIT may exert positive therapeutic effects on recurrent upper airway infections as adenotonsillar disease. Finally, some clinical observations suggested that AIT may add some benefits in the management of otitis media with effusion (OME). The results of this systematic review allow us to conclude that the efficacy of AIT in ENT disorders has been only slightly investigated and additional studies are needed.

## 1. Introduction

Allergen-specific immunotherapy (AIT) was described for the first time by Noon, who first observed the efficacy of inoculating low-dose long-term grass pollen extract in allergic patients [1]. AIT induces immune tolerance by increasing expression of allergen-specific activated Treg cells and by reducing differentiation into T helper 2 cells (TH2) [2], whose cytokines (IL-4, IL-5, IL-9, IL-13) are mediators of the allergic inflammatory response [3,4,5].

Several studies have demonstrated that both current subcutaneous (SCIT) and sublingual specific immunotherapy (SLIT) are effective in treating AR compared to placebo. The main difference between the two administration methods is the safety profile, due to the higher possible risk of side effects with SCIT [6,7,8,9,10,11,12,13,14,15,16,17,18,19,20,21,22,23,24,25,26,27,28,29]. Thus far, AIT is recommended in the long-term management for allergic rhinitis (AR) and asthma, representing the only disease-modifying treatment [30,31,32,33,34]. Literature data, in fact, demonstrated that AIT reduces AR symptoms and decreases the use of symptomatic therapies over the years [35,36,37].

Furthermore, numerous randomized clinical trials have shown a persistent benefit from AIT in adults up to 3–5 years after the end of therapy [35,36,37]; contrarily, in the pediatric population the efficacy over time has yet to be confirmed [38,39,40]. Finally, the reduced risk of sensitization to new allergens achieved with AIT is still a matter of debate [35,36,37]. In the ear, nose, and throat (ENT) field, AIT is currently indicated for AR patients who had inadequate benefits from standard therapy. Several studies have also been conducted to evaluate the efficacy of AIT on different ENT manifestations, in which allergy might play a role in the etiopathogenesis, as local allergic rhinitis (LAR), rhinosinusitis (RS), otitis media (OM), and adenotonsillar (AT) disease [18,19,20]. Thus far, the management of allergy in these diseases and the indication to a specific AIT is still an object of debate and there is still a lack of systematic reviews and meta-analyses evaluating the efficacy of AIT on ENT diseases aside from AR [41].

This systematic review aimed to evaluate the potential role of AIT in treating different ENT disorders in which its efficacy is still debated and specifically on local allergic rhinitis (LAR), acute (ARS) and chronic rhinosinusitis (CRS), adenotonsillar diseases (AT), and otitis media (OM).

## 2. Materials and Methods

### 2.1. Search Strategy

This systematic review was conducted in accordance with the Preferred Reporting Items for Systematic Review and Meta-Analysis (PRISMA) [42] process to identify published experimental and clinical articles about AIT and ENT diseases published from 1980 to 2022. Literature searches were performed, and 3014 manuscripts were screened primarily by Ovid Medline and EMBASE and from other sources (PubMed Central, Cochrane review, Web of Science, and Google Scholar).

We performed two different searches using MeSH-terms. The first group of authors focused on clinical studies on the efficacy and safety of AIT in LAR matching the term as follows: [(local allergic rhinitis) OR (LAR) OR (local atopy) OR (local respiratory allergy) OR (entopy)] AND [(immunotherapy) OR (AIT) OR (allergen-specific immunotherapy) OR (desensitization) OR (subcutaneous specific immunotherapy) OR (sublingual specific immunotherapy) OR (SLIT)]. The second group of authors focused on clinical studies on the efficacy and safety of AIT in acute and chronic rhinosinusitis matching the term as follows: [(respiratory infections) OR (acute rhinosinusitis) OR (chronic rhinosinusitis) OR (sinusitis) OR (rhinosinusitis) OR (recurrent sinusitis) OR (endoscopic sinus surgery) OR (URTI) OR (upper recurrent respiratory infection) OR (common cold)] AND [(immunotherapy) OR (AIT) OR (allergen-specific immunotherapy) OR (desensitization) OR (subcutaneous specific immunotherapy) OR (sublingual specific immunotherapy) OR (SLIT)]. The third group of authors focused on clinical studies on the efficacy and safety of AIT in adenotonsillar diseases (AT) and otitis media (OM) matching the term as follows: [(adeno-tonsillar disease) OR (tonsillitis) OR (adenoiditis) OR (adeno-tonsillar hypertrophy) OR (Eustachian tube disfunction) OR (middle ear cytokine) OR (middle ear inflammation) OR (acute otitis media) OR (chronic otitis media) OR (otitis media with effusion) OR (recurrent acute otitis media) OR (middle ear effusion) OR (chronic suppurative otitis media) OR (otitis)] AND [(immunotherapy) OR (AIT) OR (Allergen-specific immunotherapy) OR (desensitization) OR (subcutaneous specific immunotherapy) OR (Sublingual specific immunotherapy) OR (SLIT)].

### 2.2. Study Selection

In the first screening, the authors read the title and abstract of articles being as inclusive as possible. The abstracts were screened by independent reviewers. Any disagreement was solved by consensus. Inclusion and exclusion criteria were identified before the selection of relevant studies. The inclusion criteria were: primary research (including descriptive studies, observational studies, randomized trials, and basic science articles), published after 1980, addressing AIT and local allergic rhinitis (LAR), acute and chronic rhinosinusitis (ARS and CRS), adenotonsillar diseases (AT), and otitis media (OM). We excluded secondary research studies (e.g., review articles or systematic review), case studies, newspaper articles, lectures, letters, comments, personal narratives, consensus conferences, and editorials. Only articles with full text available were included. Additional studies were manually identified from the reference lists of retrieved literature.

We excluded all the articles that did not meet the inclusion criteria or deal directly with the issue investigated.

## 3. Results and Discussion

**Figure 1:**
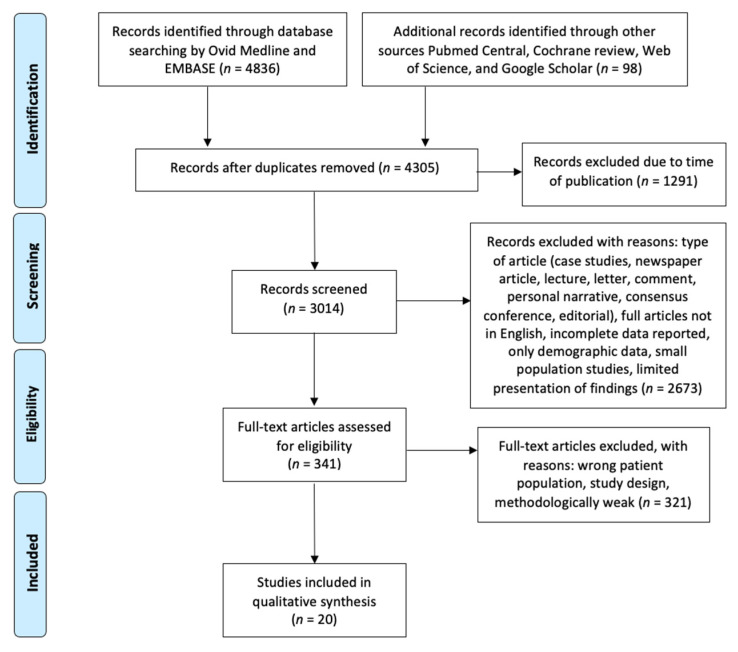
Prisma flow diagram of the systematic search.

Results of the systematic search are shown in Figure 1. Our search yielded a total of 4305 articles after duplicates removal. We excluded 1291 articles due to year of publication and 3014 records were therefore screened. Due to the type of article, wrong population, and study design, 2994 articles were excluded. This resulted in 20 publications that were finally included. We summarized in tables the included studies classifying evidence using GRADE methodology and quality by Black and Downs method [43]. No studies were included in a quantitative synthesis (meta-analysis).

### 3.1. Efficacy of Allergen-Specific Immunotherapy in the Treatment of Local Allergic Rhinitis

Local allergic rhinitis (LAR) is typically characterized by the presence of classic AR symptoms, but negative skin prick test (SPT) and undetectable serum specific IgE (sIgE) against inhalant allergens [44,45]. According to recent research, LAR might affect more than 45% of patients previously diagnosed with non-allergic rhinitis (NAR) [46]. LAR seems to be characterized by a TH2 inflammatory response in nasal mucosa during natural exposure to aeroallergens with the release of mediators from mast cells, eosinophils, B cells, and T cells, and a positive response to a nasal allergen provocation test (NAPT) with local production of IgE, tryptase, and eosinophil cationic protein (ECP) in the absence of systemic atopy [46,47,48]. However, TH2 inflammation is not the only causative mechanism in all AR. Innate immunity, represented by the group 2 innate lymphoid cells (ILC2s), is also involved in house dust mite-induced AR, where AIT has modest effects.

Innate lymphoid cells (ILCs) that derive from common lymphoid progenitors with TH2 have recently been discovered. They contribute to immunity through the secretion of signaling molecules and the regulation of innate and adaptive immune cells. AR is the archetype of the Th2-acquired immune reaction and the efficacy of AIT, particularly for pollen allergens, is consistent with this acquired Th2 immunodominant role. However, ILC2 may also be involved, possibly depending on the allergen. For example, they increased in the blood of house-dust-mite (HDM)-sensitive AR patients, but not in sensitivity to mugwort, compared with healthy controls. ILC2 also increased during the grass pollen season in AR subjects compared to controls, apparently abrogated by SLIT with grass pollen. The number of ILC2 circulating in the blood also increases following the cat allergen challenge. Therefore, while AR remains the archetype of Th2 disease, a contribution from ILC2 seems likely, perhaps more prominent in HDM-AR than in pollen-AR [49]. Thus, in contrast to the dominant role of AIT in Th2-acquired immunity, remarkably in pollen-induced AR, AIT has modest effects on ILC2s-influenced AR. Therefore, this indirect evidence may elicit the lesser degree of benefit of AIT for LAR with house-dust-mite allergens [41]. Similar to AR, LAR can be classified as seasonal-perennial, intermittent-persistent, and mild, moderate, and severe [48,50], and some studies found the transformation from LAR to AR after years of follow-up [41].

Moreover, LAR worsens over time, with impairment in quality of life (QoL), persistence, and increasing severity of rhinitis, and associations with conjunctivitis and asthma [50,51].

The first-line therapy for LAR is represented by nasal corticosteroids with/without antihistamines as well as allergen avoidance measures. However, these treatments are unable to disrupt the natural progression of the disease towards symptoms worsening [51,52]. Since both AR and LAR share many clinical and pathophysiological features, including eosinophilia, it is reasonable to evaluate the potential role of allergen immunotherapy in LAR patients [52,53].

A pilot observational study compared the safety and efficacy of preseasonal grass SCIT in patients with moderate-severe seasonal LAR due to grass pollen [54]. The group receiving SCIT showed a significant improvement in nasal tolerance with significantly higher threshold concentrations of grass pollen in nasal allergen provocation tests (NAPTs). SCIT with grass pollen was shown to be safe and effective in patients with LAR by improving objective and subjective parameters.

In a randomized, double-blind, placebo-controlled phase II trial, the same investigator group established the role of immunotherapy in LAR [55]. Thirty-six LAR patients received Pangramin PLUS *Dermatophagoides pteronyssinus* (DP) or placebo for 24 months. Allergen immunotherapy for DP produced significant improvement. Immunotherapy was well tolerated, and no systemic reactions were reported.

In another randomized, double-blind, placebo-controlled study, 56 patients with moderate-severe LAR to grass pollen received *Phleum pratense* (Phl)-SCIT with a depigmented polymerized pollen vaccine or placebo for the first year, and Phl-SCIT the second year [56]. The study showed that Phl-SCIT had a short-term and sustained effect with significant improvements of all clinical outcomes and QoL score. Phl-SCIT significantly increased serum sIgG4 levels and allergen tolerance, from the 6th to 24th month of treatment. At the end of the study, 83% of patients treated with SCIT over 6 months tolerated a concentration of Phl pratense around 50 times higher than baseline, and 56% had a negative NAPT. The trial suggested that SCIT with depigmented polymerized allergen extracts was a safe and clinically effective treatment for LAR to Phl pratense.

Moreover, a randomized, double-blind, placebo-controlled trial by Bożek et al. conducted on 28 LAR patients treated with birch SCIT [57] showed a significant decrease in symptom medication score, up to 65% compared to baseline, an increase in immunoglobulin G4, and a decrease in nasal-specific IgE in the AIT group, compared to the placebo group. The study demonstrated that AIT for birch pollen was clinically effective and exhibited good tolerance in patients with LAR.

In a recent prospective, double-blind, and placebo-controlled trial, SLIT tablets improved nasal and bronchial symptoms and reduced symptomatic treatment in a small sample size of 17 adult patients with LAR and asthma and with hyperresponsiveness to house dust mite (HDMs) [58].

Accordingly, Yin et al. [59] demonstrated a significant decrease in nasal sIgE after 3-year treatment with SLIT of *Dermatophagoides farinae* drops in 60 patients with LAR. However, the efficacy of SLIT in patients with LAR is still debated and high-quality studies are required [41].

Table 1 resumes the above studies suggesting that AIT might modulate the immune mechanisms underlying LAR and have beneficial and safe effects for LAR. Literature data confirmed the hypothesis that AIT may be considered an effective therapeutic option in LAR patients although its effects are restricted to studies with short-term follow-up. These short-term benefits included the improvement of both symptoms and disease specific QoL. However, the evidence of AIT effectiveness in long-term follow-up and in the pediatric population is still lacking.

### 3.2. Efficacy of Allergen-Specific Immunotherapy in Acute and Chronic Rhinosinusitis

Rhinosinusitis (RS) is a common inflammatory disease affecting both the mucosa of the nose and paranasal sinuses with varying degrees of pathological involvement and clinical presentation [60]. Herewith, we investigated the available literature evidences to verify the hypothesis that AIT may offer advantages in the management of these conditions.

#### 3.2.1. Efficacy of Allergen-Specific Immunotherapy in Acute Rhinosinusitis

The role of allergy in acute RS (ARS) is a matter of debate, with literature both supporting and discouraging it as a predisposing factor for ARS. The link between AR and ARS has been proposed through several pathologic mechanisms, including reduced mucociliary clearance, mucosal swelling, and narrowing of sinus ostia. However, AR’s contribution to the development of ARS, or as a disease modifier, is still unclear. Some studies demonstrated an increased risk of ARS episodes both in adults and children affected by AR compared to healthy controls [61,62,63,64,65,66]. Conversely, a review by Pant et al. concluded that insufficient evidence exists to confirm AR as a significant predisposing factor for ARS, and Frerichs et al. reported that no evidence supports a prolonged course of ARS in the setting of AR [67,68]. A recent prospective study in children demonstrated that although ARS was common in the studied population, the most common risk factor for ARS was an acute viral infection and there was no difference in incidence between allergic and non-allergic patients [69]. Moreover, there are no studies in the literature evaluating the role of allergy in recurrent ARS. In this scenario, no significant data supports the role of AR as a risk factor or disease modifier for the development of ARS and, similarly, there is a lack of literature to support the use of AIT in ARS.

#### 3.2.2. Efficacy of Allergen-Specific Immunotherapy in Chronic Rhinosinusitis

Allergy has been suggested to be involved in CRS with nasal polyps (CRSwNP) pathogenesis since atopic disease and eosinophilic inflammation are strongly associated with a TH2-mediated response, that widely represents CRS’s preponderant endotype, especially in Western countries [70,71,72]. Furthermore, the association between AR and CRS has been described a long time ago, but the literature lacks firm and evident conclusions regarding the role of allergy in CRS, and pathogenic mechanism remains controversial both in CRSwNP and CRS without nasal polyps (CRSsNP) [66,67,68,73]. Several studies showed that atopy is more prevalent in CRS populations, especially in patients with refractory CRS. The prevalence ranges from 50 to 84%, with most patients being sensitized to multiple allergens. Some studies showed that perennial allergens may play a more significant role, indicating that specific treatment might improve CRS outcomes [74].

DeYoung et al. conducted a systematic review about the efficacy of AIT in CRS. They found that symptom scores, endoscopic scores, and CT scores generally improved in patients treated with AIT compared to baseline data and to control patients over the short term. They also noted a decreased need for revision surgeries, interventional office visits, and intranasal and oral steroid use. According to the authors, AIT has a potential role of long-term modulator of the CRS underlying immune dysfunction. Although the improvement in CRS symptoms might only reflect the treatment of AR, the advantages of AIT would be appreciable to patients regardless of the etiology. However, they concluded that there is weak evidence to support the use of AIT as an adjunctive treatment for patients with CRS. Nevertheless, their observations were limited by the little data available on a very heterogeneous group of diseases including CRSsNP, CRSwNP, and allergic fungal rhinosinusitis subgroups. Thus, the authors suggested the need for prospective, randomized, placebo-controlled trials to confirm the specific benefits of AIT in atopic CRS [75]. A very recent systematic review by Borish et al. [76] on AIT to aeroallergens for CRS identified four studies between 1983 and 2004. Two prospective trials included adults with CRSwNP and CRSsNP following surgery [77,78], and one studied children with CRS (polyp status not stated) without previous surgery [79]. While the study of Nishioka on CRSwNP adults (*n* = 72; 66 on SCIT and 6 controls) was inconclusive regarding the therapeutic benefit of SCIT, Schlenter reported improved symptom scores for patients receiving SCIT (*n* = 15) in addition to INCS in several cases, at four months and after long-term follow-up associated with improved radiologic outcome scores compared to controls [77,78].

In the pediatric study, 20 patients treated with SLIT experienced fewer rhinitis symptoms, less turbinate swelling, and improved radiologic findings than controls [79].

A retrospective, uncontrolled case series of 114 CRSsNP patients treated with SCIT showed improvement in outcome questionnaire scores, decreased sinus pain, discolored mucus discharge, and nasal congestion along with a 54% reduction in sinus surgeries [80].

Recently, Jia et al. published a prospective single-center study conducted on 64 CRS and AR patients after endoscopic sinus surgery (ESS) comparing the effects of the use of adjuvant AIT versus the standard of care on clinical, microscopic, and biochemical aspects. Based on the results obtained after one-year follow-up, the authors concluded that the addition of AIT improves patients’ symptoms and QoL, promotes the epithelialization of the mucosa in the surgical cavity, and adjusts the local immune response [81]. According to the authors, there is no convincing evidence supporting the hypothesis that atopy is causative in CRS, but it seems that co-existing AR accentuates type 2 inflammatory mechanisms of CRS acting as a disease amplifier [70,71,72,81,82]. Specific randomized clinical trials supporting the use of AIT as an exclusive treatment in CRS are missing. For these reasons, authors focused their attention mainly on the role of AIT as an adjuvant treatment able to improve symptoms and the surgical outcome over short-term follow-up (Table 2) [81].

Over the past few years, authors focused on the evidence that the prevalence of allergy in CRS may vary between subgroups or phenotypes, with central compartment atopic disease (CCAD) and allergic fungal rhinosinusitis (AFRS) having a stronger association than idiopathic CRSwNP and CRSsNP.

CCAD is a nasal inflammatory subtype of type 2 diffuse CRS characterized by polypoid degeneration of the central compartment of the nasal cavity, which includes either the postero-superior nasal septum, the middle turbinate, the superior turbinate, or in combination [60,83]. Overall, this central pattern of inflammatory changes is associated with 97.6% of prevalence of clinical AR [84]. Given the strong association, AIT may be a promising treatment regimen for CCAD patients. However, this subgroup has only recently been described and, for this reason, there is a poor number of studies on its medical treatment. In a retrospective analysis, Steehler et al. evaluated the outcomes of primary ESS in patients with different phenotypes of CRSwNP, among which a variable percentage of the participants received AIT. Polyp recurrence and ESS revision rates were significantly lower in CCAD than other CRSwNP subtypes, although AIT has not been closely studied with respect to outcomes and disease control [85]. According to Edwards et al., systemic allergy testing should be recommended in the workup for CCAD, and since LAR may be present in the subset of patients with CCAD, further study on the role of medical therapies and AIT should be undertaken in CCAD, in order to verify whether the latter could be a surgery-sparing treatment option or an adjunct after endoscopic sinus surgery [86].

AFRS is another distinct, often more severe, eosinophilic subtype of CRSwNP defined by specific characteristics proposed in 1994 by Bent and Kuhn including type 1 hypersensitivity, nasal polyps, specific radiological findings, eosinophilic mucus without fungal invasion, and positive fungal stain [87]. The sensitization to fungal antigens and the common finding of concomitant allergic disease in AFRS support the hypothesis that fungal and non-fungal allergen AIT may be useful in conjunction with endoscopic sinus surgery, to improve the outcome of surgery. Gan et al., in a systematic review, confirmed the potential benefit in treating AFRS with AIT [88]. Furthermore, retrospective cohort studies on post-surgical AFRS revealed improvements in endoscopic mucosal staging and reduced need for both topical and systemic steroids [89], aside from fewer office visits and revision surgery in patients treated by AIT [90]. Moreover, Mabry’s group published the results from a prospective trial, confirming the efficacy of AIT on short-term outcomes (3–4 years) and avoiding the need of surgical revision, whereas on long-term (from 4 to 10 years), AIT failed to show any additional benefit as compared to the control group, displaying that AIT loses its potency after being stopped for a longer duration [91].

In conclusion, AIT could be an adjunctive treatment for patients with CRSwNP because it might improve symptoms and the outcome of surgery over short-term follow-up. Given the minimal data and limitations to available data, there is little justification to support the use of SCIT for CRSsNP. There have been no studies of SLIT in patients with CRSsNP.

Literature data seem to confirm that AIT may be a promising treatment regimen for CCAD and it is likely to improve symptoms and to reduce revision surgery in atopic individuals with AFRS. For these reasons, although there is a lack of placebo-controlled randomized trials comparing fungal or non-fungal desensitization in AFRS, and although conclusive evidence of AIT efficacy is not present to date, EPOS 2020 steering group [59] summaries with a 2b level of evidence show that AIT in atopic individuals with AFRS is likely to improve symptoms and to reduce revision surgery.

**Table 2:** Studies on efficacy of AIT in CRS.

Authors (Years)	Type of Study	Patients (n)	Methods	Duration of AIT Therapy	Duration of FUP	Mean Outcomes Measured	Results	Conclusions	Y/N
Nishioka et al., 1994 [77]	Prospective study	72 CRS with allergy, post-surgery		Not specified	14.9 months (range 0.75–44.3 months)	Effect of AIT on middle meatotomy patency, synechiae formation, and recurrent polyps in allergic patients	AIT given either before or after surgery does not statistically influence middle meatotomy patency, synechiae formation, or recurrence of polyps after FESS	AIT does not influence the outcomes measured	N
Schlenter et al., 1983 [78]	Prospective study	65 post-surgery		N/A	N/A	Symptoms and radiographic scores	AIT group had less severe symptoms score and greater improvements of radiographic score	The treatment of allergic sinusitis with hyposensitization offers a better prognosis in the long run	Y
Asakura et al., 1990 [79]	Prospective study	52 children with CRS (no prior surgery)	Children were treated with either the combination of antigen specific immunotherapy and medication with lysozyme chloride preparation or medication alone	2–3 months	N/A	Scoring system of individual symptoms (sneezing, rhinorrhea, nasal obstruction, overall symptoms), objective signs (hypertrophy of turbinates, amount of nasal secretions), radiographic findings (XP shadow of maxillary sinus)	Symptoms and radiographic improvements were significantly better in AIT group	The addition of AIT can improve patients’ symptoms and radiographic outcomes	Y
Nathan et al., 2004 [80]	Retrospective case series	114	Patients were surveyed twice, with the first a recall of symptoms before starting immunotherapy and the second an evaluation of current symptoms	3.3 years (mean), at least 12 months	N/A	Sinusitis Outcomes Questionnaire	Mean reduction of 51% in the overall symptom score of the patients after receiving immunotherapy and 54% fewer surgeries	Immunotherapy is an effective treatment for patients with sinus disease and AR	Y
Li et al., 2021 [81]	Prospective study	64 CRSwNP with AR post-surgery	Patients were divided into three treatment groups represented by standard medication alone; standard medication and nasal irrigations; standard medication, nasal irrigations and specific subcutaneous immunotherapy	>3 years	N/A	SNOT-22, TNSS, electron microscopy inflammatory mediators (ECP, IL-8, IFN- γ, IL-25, IL-33, and IL-17)	Clinical scoring improvement, more orderly arrangement of the cilia and lower expression levels of inflammatory mediators after 1 year follow up in IT group	The addition of AIT can improve patients’ symptoms and quality of life, promote the epithelialization of the mucosa in the surgical cavity and adjust the local immune response	Y
Steehler et al., 2021 [85]	Retrospective study	132 CRSwNP subtypes post-surgery	Electronic records review of maintenance therapy for postoperative treatment, follow-up visits, pathology findings, CT imaging data, and outside records	Not specified	At least 12 months	Polyp recurrence, revision ESS, oral steroid use, oral antibiotic use	Polyp recurrence and ESS revision rates were significantly lower in CCAD than other CRSwNP subtypes in an additional AIT setting	Given the association of inhalant allergy with CCAD, AIT is a consideration in the treatment regimen for these patients	Y
Folker et al., 1998 [89]	Retrospective cohort study	22 AFRS post-surgery	Study patients were treated with specific immunotherapy fungal antigens while control group received no immunotherapy	>24 months	12–50 months (range)	CRS Survey, Kupferberg stage, corticosteroid use	AIT treated group achieved better results in all outcomes	Significant reduction in polyp reformation, corticosteroid requirements and improved quality of life in AFRS patients receiving additional AIT	Y
Bassichis et al., 2001 [90]	Retrospective cohort study	60 AFRS post-surgery	Review of the database focusing on postoperative management (nasal irrigation, intranasal steroids, systemic steroids, antibiotics, immunotherapy, in-office procedures and repeat surgery)	Not specified	48.5 months (mean)	Revision surgery, outpatient intervention	Less revision surgery (11% vs. 33%) and less clinic visits in the IT treated arm	Post-operative AIT with relevant fungal antigens is an important aspect of the treatment of AFS resulting in decrease re-operation rates and office visits requiring intervention	Y
Marple et al., 2002 [91]	Cross- sectional study of prospective protocol	17 AFRS post-surgery	Outpatient records review of follow up, SNOT-20 quality-of-life survey, ongoing medical/surgical intervention and blood immunoglobulin levels	Not specified	46–138 months (range)	Kupferberg stage, IgE levels, SNOT-20 in long term follow up	No significant difference in long-term outcomes was seen between those patients treated with fungus-specific IT and those treated with other methods	After successful initial treatment and control of AFRS, many patients can achieve a quiescent disease state	Y

Abbreviations. AIT: allergen immunotherapy; FUP: follow-up; CRSwNP: chronic rhinosinusitis with nasal polyps; SNOT-22: sino-nasal outcome test; TNSS: total nasal symptom score; ESS: endoscopic sinus surgery; CCAD: central compartment atopic disease; AFRS: allergic fungal rhinosinusitis; CR: chronic rhinosinusitis; FESS: functional endoscopic sinus surgery; AR: allergic rhinitis.

### 3.3. Efficacy of Allergen-Specific Immunotherapy in AT Diseases

Adenotonsillar tissue (ATT) seems to be directly involved in the IgE-mediated sensitization [92,93] playing a fundamental role in adaptive immune response regulation. The B cells involved in the specific immune response represent more than 50% of the ATT lymphocyte population. Furthermore, a peculiar mast cell distribution in the inter-follicular tonsillar regions in allergic subjects has been demonstrated [94].

Cho et al. reported that the sIgE-positive rate was significantly higher in local tissue than in serum, indeed, over 36% of children with sIgE-negative serum tested positive for sIgE in adenotonsillar tissue suggesting that there may be a localized allergic reaction in AT tissues even in the absence of systemic atopy in children with ATH [95]. Thus far, the relationship between AT disease and clinical manifestation of allergy, including AR, is still debated. Recently, our research group [96,97,98] performed a systematic review of the literature supporting the importance of investigating the correlation between allergy and specific phenotypes of AT disease and specifically isolated adenoid hypertrophy (AH), isolated tonsillar hypertrophy (TH), combined adenotonsillar hypertrophy (ATH), and recurrent infection of upper airway tract [97]. Concerning obstructive disease, we concluded that it was possible to establish a link only between allergy and ATH or AH, whereas some studies described a mainly negative correlation between allergy and isolated TH. Unfortunately, no manuscript has investigated the possible efficacy of AIT on these disorders. However, this is explained by the age of onset of these pathologies and the reduced indication for AIT in that age range.

It is well known that allergic children are more prone to upper airway tract infections [99]. Only few studies have investigated the possible link between allergy and AT infections, with the detection of peculiar immunological abnormalities, as impairment of human beta-defensin and S100A7 antimicrobial proteins, in the tonsils of allergic children undergoing surgery for recurrent acute tonsillitis compared to non-allergic individuals [100,101]. Based on a possible causative link between allergy and AT disease and on clinical evidence of an increased number of upper airway infections in allergic children, some authors have postulated that AIT is capable of exerting an adjunctive anti-allergic activity in AR children. [102,103,104]. This would derive from the long-lasting action of AIT on T lymphocytes resulting in both restoring Th-1 polarization to homeostasis and reducing the inflammatory reaction with an enhanced INF-γ synthesis [105]. Ciprandi et al. [102] first documented the adjunctive anti-allergic activity of two-year preseasonal pollen SLIT in 40 patients with AR, by attesting a reduced number of infections and drug prescriptions in treated patients compared to controls. These results have been subsequently confirmed by other researchers [103,104]. Occasi et al. [104] evaluated the effectiveness of SLIT on susceptibility to respiratory tract infections in 143 children receiving AIT for AR compared to 122 controls, in a six-year observation period. They found that children receiving AIT after two years of SLIT reported a lower prevalence of respiratory tract infections when compared to children not undergoing SLIT.

Barberi et al. [103] studied the impact of six-months of house dust mite SLIT in 40 children with dust mite AR. They reported a significantly reduced number of respiratory infections in treated children compared to controls, and specifically less pharyngo-tonsillitis, bronchitis, and fever episodes, and decreased medications (including antibiotics and antipyretics) consumption.

In conclusion, although it is possible to establish a link between allergy and ATH or AH, to date no manuscript has been identified that investigates the possible efficacy of AIT on these disorders, possibly due to the reduced indication of AIT in children (Table 3).

Conversely, a few studies have investigated the possible link between allergy and AT infections, concluding that AIT may exert positive therapeutic effects on allergic and extra-allergic symptoms in AT infections, reducing the number of infections and drug prescriptions in treated patients compared to controls.

### 3.4. Efficacy of Allergen-Specific Immunotherapy in Management of Otitis Media

The link between allergy and chronic or recurrent middle ear infections, including recurrent acute otitis media (RAOM) and persistent otitis media with effusion (OME), although controversial, has been long postulated; thus, atopy is counted among risk factors for acute infectious exacerbations in otitis-prone children [106]. The putative pathogenic mechanisms linking allergy and otitis are the reduced patency of the Eustachian tube due to its mucosal swelling and the reduced mucociliary clearance related to allergy, and the active immunological participation of the middle ear mucosa as a shock organ responding to antigenic stimulation. Based on this last hypothesis, the middle ear would be an integral and effective part of the united airway concept [107,108,109]. The shock organ theory is supported by the pathological evidence of increased expression of Th2-mediators into samples taken from the middle ear mucosa/fluid of atopic subjects [107,110] and of detection of antigen specific IgE into the middle ear effusion of 62% of patients with severe eosinophilic otitis media [111,112].

From a clinical point of view, Zhang et al. [113] concluded that allergy or atopy are significant risk factors for both chronic and recurrent otitis media, with a prevalence ranging between 24 and 89%. Data suggested that indoor allergens and respiratory allergies, including AR, play a role in the development of chronic/recurrent middle ear disease [113]. Conversely, Ciprandi et al. [114] failed to find a solid and inconvertible etiological correlation between allergy and middle ear disease. The authors concluded that, despite the impact of allergy on otitis media not being able to be neglected, neither routine allergy screening should be performed in children with otitis media, nor otologic assessment in allergic children, unless the coexistence of a cogent clinical evidence is present. Afterwards, several authors suggested that interesting observations may be achieved by analyzing data based on specific clinical phenotypes [99,115,116]. Torretta et al. [99] reported that atopy or allergy were significantly more prevalent in children suffering from RAOM with recurrent/persistent OME episodes compared to children with simple RAOM (i.e., respectively, atopy: 73% vs. 39%; allergy: 60% vs. 36%). De Corso et al. [116] recently pointed out that allergy is mainly linked to OME and its recurrent acute exacerbations, thus recommending to include allergy testing in the diagnostic workup of children with OME and to search for OME in children with persistent to moderate AR. Krein-Moller et al. [117] found a significant relationship between OME and AR in 291 children during their first six years of life. The authors speculated that the causative mechanism would be a diffuse allergic inflammatory reaction after antigenic stimulation rather than the sole mechanical obstruction due to nasal/Eustachian tube mucosal swelling.

Based on the linking between allergy and OME, some potential adjunctive therapeutic benefits of AIT on middle ear complaints related to OME have been identified [118,119]. Specifically, Hurst [119] in a case-series of 89 patients (including 52 children younger than 15) with intractable chronic OME or drainage from the middle ear (all proved to be atopic), found that specific AIT achieved complete (85%) or partial (5.5%) OME resolution in almost all cases; on the contrary, none of the controls had spontaneous recovery. This is in accordance with the contemporary report by La Mantia et al. [118], who documented complete OME resolution in more than 50% of children with concomitant AR receiving a two-year course of dust mites AIT, with a significantly better outcome in the study group compared to children receiving anti-allergic medications alone.

In conclusion, some clinical observations suggested the effectiveness of specific AIT on OME (Table 4). However, scientific evidence mainly derives from observational and non-controlled studies performed on children with multiple clinical manifestations of allergy; thus, additional studies are needed to confirm the efficacy of AIT in ear disorders.

## 4. Conclusions

To the best of our knowledge, this is the first systematic review that assessed the efficacy of AIT as a treatment on ENT diseases aside from AR. Literature data confirmed that AIT may be considered an effective therapeutic option in LAR patients, although its effects are restricted to studies with short-term follow-up, whereas long-term outcomes and evidence in the pediatric population are lacking. Some interesting observations have been achieved by analyzing data about AIT and CRS.

The data support the hypothesis that AIT may add some positive benefits as an adjunctive treatment, improving symptoms and outcome of surgery over short-term follow-up in CRS patients. In addition, recent research seems to suggest that AIT may be a promising treatment regimen for CCAD, and it is likely to improve symptoms and reduce revision surgery in atopic individuals with AFRS. However, more studies are needed to confirm these observations.

Regarding AT disease, while no manuscript has investigated the efficacy of AIT on AT hypertrophic diseases, few studies support the hypothesis that AIT may exert positive therapeutic effects on recurrent upper airway infections. In addition, some clinical observations suggested that AIT may add some benefits in OME management.

The results of this review let us consider that efficacy of AIT in ENT disorders has been only slightly addressed and that additional studies are needed to confirm the efficacy of AIT in some specific fields of ENT disorders. We believe that according to the precision medicine approach, which considers the individual variability of treatments and prevention, a better understanding of the effectiveness of AIT in ENT diseases may help physicians and researchers in adopting it in routine clinical practice.

## Figures and Tables

**Table 1:** Studies on efficacy of AIT in LAR patients.

Authors (Years)	Type of Study	Patients (n)	Methods	Duration of AIT Therapy	Duration of FUP	Mean Outcomes Measured	Results	Conclusions	Y/N
Rondon Cet al., 2011 [54]	Pilot, observational	20	Preseasonal grass SCIT+ rescue medication in the spring vs. rescue medication (control group)	6 months	12 months	NAPT to grass SPT, grass pollen sIgG and sIgE, symptom and medication scores, medication-free days, severity of LAR symptoms	↑NAPT, ↑ sIgG to grass pollen ↓ symptoms, ↓ rescue medication ↓ scores and severity of rhinitis in the following spring	SCIT with grass pollen showed to be safe and effective in patients with LAR; SCIT can modify the disease course with an important reduction in severity	Y
Rondon C et al., 2016 [55]	DBPC	36	Pangramin plus DP or placebo	24 months	N/A	NAPT, TdSS, TdMS, CdSMS, MFD, SPT, DP-sIgE and DP-sIgG4, adverse events	↑ NAPT, ↓ TdSS and TdMS ↑ MFD ↑ sIgG4 no systemic reactions were reported	AIT-DP is clinically effective and safe,	Y
Rondon C et al., 2018 [56]	DBPC	56	Phl -SCIT with a depigmented polymerized pollen vaccine or placebo for the first year, and Phl-SCIT the second year	24 months	N/A	CSMS during GPS, organ-specific symptoms, MFD, rhinitis severity and asthma control RQLQ, NAPT, sIgG4, safety	Significant improvements of all clinical outcomes	SCIT with depigmented polymerized allergen extracts was a safe and clinically effective treatment for LAR to Phl pratense.	Y
Bozek A et al., 2018 [57]	DBPC	28	Birch SCIT	24 months	N/A	SMS, sIgE and IgG4 and nasal -specific IgE to Bet v 1, SMS	↓ SMS ↑ Ig G4, ↓ nasal-specific IgE	AIT for birch pollen was clinically effective and exhibited good tolerance	Y
Bozek A et al., 2021 [58]	DBPC	32	12-month treatment of SLIT for HDM	N/A	N/A	TRSS, TASS, TSS, TMS, FEV1	↓ in TRSS, TASS, TSS and TMS ↑ FEV1 after 12 months of treatment	SLIT can improve nasal and bronchial symptoms and reduce symptomatic treatment in patients with LAR and asthma and with hyperresponsiveness to HDMs.	Y
Yin ZX et al., 2019 [59]	Observational	60	Sublingual immunotherapy of *Dermatophagoides farinae* drops	3 years	N/A	Symptom scores, VAS Eosinophils in nasal secretions, nasal secretions sIgE, nasal mucous membrane excitation test	Improvement of symptom and VAS scores, eosinophilia counts in nasal secretion, nasal secretions allergen sIgE test, nasal mucous membrane excitation test	Sublingual immunotherapy of *Dermatophagoides farinae* drops in nasal cavity local allergy was effective	Y

Abbreviations. AIT: allergen immunotherapy; FUP: follow-up; DBPC: double blinded placebo controlled; DBRCT: double blinded randomized clinical trial; LAR: local allergic rhinitis; SCIT: subcutaneous immunotherapy; SLIT: sublingual immunotherapy; RQLQ: Rhinoconjunctivitis Quality of Life Questionnaire; NAPT: nasal allergen provocation test; CSMS: combined symptom medication score; SMS: symptom medication score; AR: allergic rhinitis; VAS: visual analogue scale; SPT: skin prick test; HDM: house dust mite; TRSS: total rhinitis score; TASS: total asthma symptom score; TSS: total symptom score; TMS: total medication score; TdSS: Total daily symptoms; DP: *Dermatophagoides, pteronyssinus*; CdSMS: combined daily symptoms-medication score; MFD: medication free days; Phl-SCIT: *Pleum*-SCIT; GPS: grass pollen season; FEV1: expiratory flow volume in 1 s; sIgG: specific IgG; sIgE: specific IgE.

**Table 3:** Studies on efficacy of AIT in adenotonsillar disease.

Authors (Years)	Type of Study	Patients (n)	Methods	Duration of AIT Therapy	Duration of FUP	Mean Outcomes Measured	Results	Conclusions	Y/N
Ciprandi et al., 2013 [102]	Case- control	77	To investigate the impact of SLIT on extra-allergic outcomes (number of infections and drug prescriptions) in children with allergic rhinitis.	2 years	2 years	The use of drugs, the presence of respiratory symptoms and extra-allergic clinical manifestations	SLIT is effective in reducing the number of respiratory infections, drug prescriptions and improving symptoms in treated children compared to controls.	SLIT exerts adjunctive anti-allergic effects.	Y
Barberi et al., 2015 [103]	Case- control	40	To investigate the impact of a 6-months high-dose house dust mite SLIT on respiratory tract infections in children with allergic rhinitis.	6 months	N/A	The number of respiratory infections (acute rhinosinusitis, otitis, pharyngotonsillitis, laryngitis, bronchitis, pneumonia), the presence of fever, snoring, the use of antibiotics, anti-inflammatory drugs, oral corticosteroids and fever-reducers	A significantly reduction in the number of respiratory infections (i.e., pharyngo-tonsillitis, bronchitis, fever episodes), and in antibiotics/antipyretics prescriptions was documented in the study group compared to the control one.	A short course of SLIT could reduce the number of respiratory tract infections in allergic rhinitis children.	Y
Occasi et al., 2015 [104]	Case- control	265	To evaluate the effectiveness of SLIT on susceptibility to respiratory tract infections in children with allergic rhinitis.	2 years	6 years	The number of respiratory tract infections	The number of respiratory tract infections was significantly reduced in the study group compared to the control group during the last two years of the treatment; no differences were detected before.	SLIT could have possible benefic effects on respiratory infections	Y

Abbreviations. FUP: follow-up; SLIT: sublingual immunotherapy.

**Table 4:** Studies on efficacy of AIT in otitis media with effusion.

Authors (Years)	Type of Study	Patients (n)	Methods	Results	Duration of AIT Therapy	Duration of FUP	Mean Outcomes Measured	Conclusions	Y/N
La Mantia et al., 2021 [118]	Case- control	40	To investigate the effectiveness of 2-year dust mite immunotherapy in children with allergic rhinitis and otitis media with effusion.	Complete recovery was attested in more than 50% of treated children compared to 15% of children receiving anti-allergic treatment alone.	2 years	18 months	Change in tympanometry findings	OME has an allergic background, and it could effectively respond to specific AIT.	Y
Hurst, 2008 [119]	Cross sectional	52	To assess the therapeutic benefit on middle ear complaints deriving from specific allergen immunotherapy.	An atopic status was discovered in 100% of patients. Complete/partial recovery was attested in more than 95% of treated patients.	4 years (as a mean)	2–8 years (range)	Resolution of OME (documented by means of pneumatic otoscopy, tympanometry, audiometry); episodes of acute ear discharge; needing for tympanostomy tube placement	OME is an immune mediated allergic condition responding to specific AIT	Y

Abbreviations. FUP: follow-up; OME: otitis media with effusion; AIT: allergen immunotherapy.

## Data Availability

Data are available upon reasonable request.
